# Adherence of Healthcare Workers to Saudi Management Guidelines of Heat-Related Illnesses during Hajj Pilgrimage

**DOI:** 10.3390/ijerph18031156

**Published:** 2021-01-28

**Authors:** Yasir Almuzaini, Nour Abdulmalek, Sujoud Ghallab, Abdulaziz Mushi, Yara Yassin, Saber Yezli, Anas A. Khan

**Affiliations:** 1 Global Center for Mass Gatherings Medicine, Ministry of Health, Riyadh 12341, Saudi Arabia; Nabdulmalek@moh.gov.sa (N.A.); dr.sujoudghallab@yahoo.com (S.G.); abhmashi@moh.gov.sa (A.M.); yara@yassin.com (Y.Y.); SYezli@moh.gov.sa (S.Y.); anaskhan@ksu.edu.sa (A.A.K.); 2 Department of Emergency Medicine, College of Medicine, King Saud University, Riyadh 12372, Saudi Arabia

**Keywords:** national guideline, adherence, healthcare workers, heat-related illness, Saudi Arabia

## Abstract

Heat-related illnesses (HRIs), such as heatstroke (HS) and heat exhaustion (HE), are common complications during Hajj pilgrims. The Saudi Ministry of Health (MoH) developed guidelines on the management of HRIs to ensure the safety of all pilgrims. This study aimed to assess healthcare workers’ (HCWs) adherence to the updated national guidelines regarding pre-hospital and in-hospital management of HRIs. This was a cross-sectional study using a questionnaire based on the updated HRI management interim guidelines for the Hajj season. Overall, compliance with HE guidelines scored 5.5 out of 10 for basic management and 4.7 out of 10 for advanced management. Medical staff showed an average to above average adherence to pre-hospital HS management, including pre-hospital considerations (7.2), recognition of HS (8.1), case assessment (7.7), stabilizing airway, breathing, and circulation (8.7), and cooling (5). The overall compliance to in-hospital guidelines for HS management were all above average, except for special conditions (4.3). In conclusion, this survey may facilitate the evaluation of the adherence to Saudi HRIs guidelines by comparing annual levels of compliance. These survey results may serve as a tool for the Saudi MoH to develop further recommendations and actions.

## 1. Introduction

In 2009, the World Health Organization (WHO) recognized climate change as one of the top five environmental causes of death worldwide [1]. Extreme weather patterns have resulted in various illnesses and mortalities around the world. Exposure to extreme heat, in particular, is a natural hazard that can affect human health and is significantly linked to the occurrence of heat-related illnesses (HRIs) [2,3]. A wide range of health conditions may result from HRIs, from mild symptoms to life-threatening manifestations such as heatstroke (HS), heat exhaustion (HE), and heat cramps [4,5]. Age, urbanization, obesity, comorbidities, lack of adaptive cooling measures, duration of exposure to heat, and relative humidity are individual and environmental risk factors that may increase the frequency and intensity of HRIs [6,7]. Patients with HRIs can present with wide clinical spectrums such as fatigue, vomiting, fainting, hyperthermia, neurological disturbance, circulatory collapse, and multiorgan failure [8]. The diagnosis of HRIs is usually based on the history of exposure to a hot environment, clinical symptoms, and signs of dehydration [9].

The Holy City of Makkah is the host of two Islamic rituals, namely Hajj and Umrah. Makkah is located in the western region of the Kingdom of Saudi Arabia (KSA), and it is characterized by a desert climate with extreme heat during the daytime. HE and HS are the leading causes of morbidity and mortality when the pilgrimage season to Makkah enters the hot cycle of the year [10,11]. For instance, in Hajj 2016, 267 pilgrims suffered from HRIs, with a mortality rate of 7.1% among patients with HS [12]. Saudi Arabia has a history of comprehensive strategies for the prevention and management of HRIs. A study conducted in 1987 indicated that during the 1980s, the government used the educational system to inform the public about heat illnesses and the strategies used to prevent morbidity and mortality [13]. In 2016, the Saudi Ministry of Health (MoH) published guidelines on the management of heat illnesses [14]. The Saudi MoH developed updated interim guidelines for HCWs in 2019; however, the guidelines are yet to be published publicly [15]. This updated guideline provides an evidence-based pathway for pre-hospital and in-hospital management of HRIs. The guideline focuses on recognition of HRIs, assessment of responsiveness and stabilization of the patients in the pre-hospital setting, measures of proper cooling in the pre-hospital setting, transfer to hospital, confirmation of diagnosis, cooling in hospital setting, and supportive therapy for multiple organ system dysfunction.

The adherence of healthcare workers (HCWs) to clinical guidelines is the cornerstone for optimal management of health disorders. Poor compliance to clinical guidelines was reported to be associated with unnecessary utilization of healthcare resources, delay in diagnosis or misdiagnosis, inadequate treatment prescription, and suboptimal clinical outcomes [16]. Many barriers were reported for optimal adherence to clinical guidelines, including the complexity of the guideline itself, inadequate knowledge and awareness, and work overload [16]. In the setting of huge mass gathering events such as the Hajj rituals, HCWs can find it challenging to adhere to all guidelines’ instructions. Thus, auditing HCWs’ adherence to the national guidelines is critical to reflect the compliance of the HCWs, barriers of non-compliance, and measures to improve guideline adherence [17]. Clinical audit is a quality improvement process that aims to improve patient care and outcomes through a systematic review of care against recognized standard criteria. Where indicated, changes are implemented, and further monitoring is used to confirm improvement in healthcare delivery [18]. The clinical audits aim to highlight the discrepancies between the actual and standard practices to identify the changes needed to improve the quality of care [19]. The Saudi MoH established the clinical audit program in 2009 to audit clinical and operational performance. It seeks to bring health service standards up to the world’s highest levels [20]. This study aims to audit the implementation of the updated 2019 guidelines for the management of HRIs focusing on HS and HE through the pre-hospital clinical pathway documented data and the observation of the case management in the heat units during Hajj. 

## 2. Materials and Methods

### 2.1. Study Design, Setting, and Population 

The Saudi government provides free health services during Hajj rituals through 16 hospitals and 128 primary health centers (PHCs), including seven seasonal health facilities. More than 13,000 core HCWs operate seasonal health facilities during the pilgrimage [21]. Besides, 60% of the countries sending pilgrims for the Hajj have medical missions accompanying pilgrims and provide them with health services during the pilgrimage [21].

This study was a cross-sectional study using a data collection form based on the updated 2019 HRI management interim guidelines for Hajj. The management guidelines were distributed beforehand to all hospitals and PHCs in the holy sites. The objective of distributing the guidelines was to familiarize all involved healthcare personnel with the diagnosis and management pathway of HRIs, focusing on HS and HE.

HRIs data were collected for all patients presenting with HRIs at the eight hospitals in the holy sites. A team of two was deployed in each hospital (total number of data collectors = 16) to collect data through a pathway checklist questionnaire by reviewing patients’ medical records. Patients included in our survey were male and female adult pilgrims (>18 years of age) who were diagnosed with HS or HE and admitted to a designated hospital during the study period. We excluded pilgrims below 18 years, patients not admitted to the hospital, and patients who were not diagnosed with HS or HE.

### 2.2. Data Collection 

Based on the interim guidelines, a data collection checklist was designed to be aligned with the clinical pathway of HRIs case management. The checklists were filled out by recruited personnel trained on the study tool and the study’s methodology. In-hospital case management data was collected in the heat unit under the supervision of the research team. The data were collected from the patients’ medical records once admitted to the heat unit and categorized into the following four sections: (1) demographic characteristics (age, gender, nationality, the residence of the pilgrim, country of origin, and hospital medical record number), (2) HE case management, (3) pre-hospital HS case management, and (4) in-hospital HS case management. The pre-hospital and in-hospital sections consisted of binary questions (Yes/No) assessing whether each patient was managed according to the recommended pathway or not (Supplementary File S1). There was incomplete data documentation in some records, which led to missing data in some of the variables in our report.

### 2.3. Data Analysis and Scoring System

All data analyses were done using SPSS 23.0 (SPSS Inc., Chicago, IL, USA). Variables were characterized using frequencies and mean values for the respective categorical and continuous variables. A scoring system was developed to identify three major themes in the clinical pathway: (1) HE case management, (2) pre-hospital HS case management, and (3) in-hospital HS case management. In addition to the three major themes, we included several subthemes under each theme. A score of 1 was assigned for each indicator/variable consistent with the HRI management interim guidelines for Hajj season 2019 (latest version during the study period) and 0 for inconsistency with the guidelines. The indicator sub-score (X) for guideline consistency was obtained as follows:

**X** = [Number of cases consistent with guidelines/Total number eligible of cases] × 10

The guideline compliance scores were divided into four categories to assess the level of compliance among pilgrims in each theme/subtheme. These categories were: poor (score < 2.5), below average (2.5 ≤ score < 5), average (5 ≤ score < 7.5), and above average (score ≥ 7.5).

## 3. Results

### 3.1. Characteristics of the Study Population 

HRIs data were collected from all eight hospitals in the holy sites from a total of 98 patients. Table 1 presents the demographic characteristics of the study population. The mean age of the study population was 58 ± 14.1 years. The male:female ratio was nearly 1:1. The majority of patients (55.3%) were ≥60 years of age. Almost half of the patients (49%) were diagnosed with HE, while the rest were diagnosed as HS cases. The vast majority of participants (93.1%) were from outside Saudi Arabia. Nationalities from the Eastern-Mediterranean region and the South-East Asia region represented more than three-quarters of the study population (46.9% and 31.3%, respectively). 

The mean age of patients diagnosed with HE was 56.3 years (standard deviation (SD) = 14.1 years, range 22–100 years). However, the mean age of the patients diagnosed with HS was 59.9 years (SD = 14.0 years, range 31–97 years). More than half of the responders suffering from HRIs were from the elderly population. Residents of Saudi Arabia suffering from HRIs were a minority in this study, accounting for 11.1% of patients with HE and only 2.4% of patients with HS (Table 1).

### 3.2. Heat Exhaustion Management Guideline Compliance Scores 

The HE management guidelines compliance scores across the two identified themes are presented in Table 2. Out of a maximum score of 10, the overall guideline compliance score was considered average in basic case management, being 5.5, while it was below average for advanced case management, being 4.7. The highest compliance sub-score of the basic case management theme was “moving patients to a cooler place”, which was considered above average (9.3). While the lowest sub-scores were observed in the “patient placed in the supine position and elevate legs and hips” and “patient started oral hydration”, being 3.8 and 2.8, respectively. Meanwhile, the highest sub-score for advanced case management was “intravenous fluids given in patients with nausea”; however, the compliance score was only average (5.5). The rest of the compliance scores in advanced case management were below average, with the lowest sub-score observed in the “cooling and transferring the patient to a health facility”, being 4.3 (Table 2).

### 3.3. Heatstroke Pre-Hospital Management Guideline Compliance Scores 

The HS pre-hospital management guidelines compliance scores across the five identified themes are presented in Table 3. Out of a maximum possible score of 10, the overall guideline compliance score for pre-hospital considerations was 7.2, which is considered average. The body substance indication was considered in 8.16% of the patients, while full pre-hospital recording was considered in 69.4% of the patients. The guideline compliance score for pre-hospital recognition of HS was 8.1, which is considered above average. Regarding the overall compliance of HCWs to pre-hospital HS case management, the compliance scores for case management (7.7) and airway, breathing, and circulation (ABC) stabilization (8.7) were above average, while the compliance cooling was average (5; Table 3). 

### 3.4. Heatstroke In-Hospital Management Guideline Compliance Scores 

The HS in-hospital management guidelines compliance scores across the five identified themes are presented in Table 4. Out of a maximum score of 10, the overall guideline compliance score for the in-hospital considerations was considered above average (7.6). Physicians adhered to appropriate personal protective equipment (PPEs) during the management of all patients, and 75.5% of the patients did not receive antipyretics. On the other hand, the compliance score to the diagnosis confirmation by rectal thermometer was above average (7.5). The majority of compliance scores to guidelines were above average, except for the overall compliance to special considerations, which was below average with a score of 4.3. For example, rhabdomyolysis was considered in 18.4% of the cases, and volume expansion was considered in 40.8% of the cases. 

## 4. Discussion 

HRIs are regularly reported during the Hajj pilgrimage, resulting in significant morbidity and mortality among pilgrims [10,12,22]. Adequate pre-hospital and hospital management is the cornerstone to ensure optimal outcomes in patients with HRIs. Thus, Saudi MoH developed comprehensive guidelines for the management of HRIs during Hajj season. Nonetheless, HCWs can find it challenging to adhere to all guideline’s instructions, especially during mass gathering events such as the Hajj rituals. Hence, it is crucial to study HCWs’ compliance with these guidelines to apply the necessary changes, which would improve the quality of health services and management. This study assessed healthcare providers’ level of compliance with the interim Saudi national guidelines for HRI management during the Hajj season 2019. 

The results demonstrated that HCWs had an average and below-average compliance to the guidelines concerned with the basic and advanced management of HE, respectively; notably, starting oral hydration, patients positioning, and transferring the patient to a health facility had the lowest compliance scores. On the other hand, HCWs’ compliance levels to the pre-hospital considerations, recognition, and HS management were average to above average. Similarly, the HCWs achieved average to above-average compliance with in-hospital considerations, diagnosis, and management of HS. However, the HCWs’ level of compliance to special considerations during HS management (such as the presence of rhabdomyolysis, hyperkalemia, and volume expansion) was below average.

Although the primary management lines for HE are simple and imply mainly proper cooling and hydration, improper management of HE can lead to devastating consequences such as HS, multiorgan failure, and death [23]. In the case of HE, moving the patient to a cooler environment such as a shaded area or an air-conditioned car, coupled with lightening the patient’s clothing, would be the first step of patient treatment [24]. In this report, moving patients to cooler places was the only management step with an above-average adherence from HCWs. This step was applied to most patients since it is the easiest and fastest step. Compliance with lightening up clothes was average due to the nature of clothes male pilgrims wear (Ihram), which are two loose pieces of cloth worn during the rituals.

On the other hand, excessive loss of water, salt, or both may increase the risk of heat illnesses. A previous study reported that on-the-spot oral hydration is an essential step for the patient treatment process [24,25]. However, starting oral hydration and placing patients in supine positions with elevated hips and legs had the lowest compliance scores. Such low compliance levels could be due to several reasons: the lack of proper knowledge among HCWs concerning the importance of proper hydration during HE attacks can represent an important reason for this inadequate adherence. Environment-related factors, including the presence of other critically ill patients needing more attention [16], diversity of cultures, and different languages could have made it more difficult for HCWs to precisely follow all the guidelines. Other patient-specific factors include the stable general condition of most patients or improvement after initial management steps. These factors could also be the reason for the below-average adherence to advanced case management of HE, including transferring patients to health facilities and considering other diagnoses in cases not improving. We also observed incomplete data documentation in the adherence to intravenous (IV) fluid administration in nauseated patients. 

On the contrary, higher compliance scores were recorded in the pre- and in-hospital management of HS. Better awareness of medical staff to the serious risks and rapid complications of HS could be a reason for this difference in compliance. The average compliance to pre-hospital considerations reflected the fair knowledge of HCWs of the proper preparation and relevant history taking for patients with HS. One of the positive outcomes of this study was the high levels of recognition and adherence to the assessment of cases with HS. All healthcare professionals should direct all their efforts to stabilize the patient’s airway, breathing, and circulation (ABC) before proceeding to more specific cooling therapy [26,27,28]. When assessing medical staff’s adherence to the ABC stabilizing guidelines, HCWs showed high adherence to the guidelines, where 87% of the eligible cases were adequately stabilized. Even after hospital admission, staff showed outstanding compliance levels to airway stabilization and maintaining oxygen saturation above 94. This relatively high adherence could result from the adequate training and readiness of medical staff for emergency scenarios related to HRIs.

On the other hand, previous studies highlighted the importance of initiating external cooling on-site and continuing cooling during transportation to medical facilities. Continuous cooling significantly improves the outcome of HS management in the pre-hospital setting [26,27]. The current study showed average compliance in the overall pre-hospital cooling process. There was a below-average adherence to both immediate and continued cooling; however, HCWs adhered to the immediate transfer and cooling methods. This variation in compliance to pre-hospital cooling could reflect the need for more healthcare training about the importance of cooling. Factors like the distance to the HS unit, transportation availability, access to cooling tools, and overcrowded environment may affect the proper adherence to the cooling process [29]. Future surveys should closely monitor all data related to the adherence to this simple yet life-saving step. Adherence to in-hospital cooling was different from pre-hospital cooling. This study showed outstanding compliance with continuous cooling and temperature monitoring. The well-organized system and fairly equipped setting of HS units and healthcare facilities is another factor to account for when comparing the cooling process pre- and in-hospital. 

This study showed high levels of adherence of HCWs to the Saudi in-hospital management guidelines of patients with HS. The use of PPE is essential for protecting both HCWs and patients from transmissible pathogens [30]. All medical staff (100%) adhered to the appropriate PPEs. The high compliance scores for using proper PPEs may be partly related to the high probability of infection transmission during a mass gathering and the rigorous measures taken by the KSA MoH on the importance of safety regulations despite dealing with a large number of patients. Training and educating medical staff on proper PPE utilization remains a critical method to maximize compliance [31].

Notably, medical staff showed an average adherence to full report taking before hospital admission. This could be due to the overcrowded nature of healthcare facilities during the Hajj season. HCWs need to be aware of the importance of the proper collection of patients’ data. Digitalization of report taking can provide faster and more efficient solutions for the report taking during the excess flow of patients with HRIs [32]. 

Rectal temperature is considered the golden standard in hospital settings to confirm the diagnosis of HS [26,28,33]. Our study results showed that 75% of the eligible heatstroke patients were diagnosed by a rectal probe, reflecting a high level of adherence to this diagnostic step. However, we observed a below-average adherence to maintaining skin temperature above 30 °C, which may reflect the need for closer follow-up to avoid the complications of induced hypothermia, including arrhythmias and coagulopathies [34]. Rectal temperature measurement is a more accurate, practical, and reliable method compared to axillary, temporal, aural, and oral thermometry [34]. 

Previous studies suggested benzodiazepines as the treatment of choice for cases of HRIs associated with seizures [35,36]. Therefore, prescribing benzodiazepines as a treatment was implemented in the Hajj interim HRI guidelines. Similarly, in the current study, we found that 6 out of 7 eligible cases were treated with benzodiazepines, which reflects a high level of compliance with the guidelines and confidence in managing this neurological complication. Healthcare professionals were also highly compliant with Saudi guidelines in providing supportive care administration for cases with organ dysfunction. However, poor to below-average compliance levels to other special considerations in HS management were observed. This could also be due to common negligence of these steps, especially in conditions that may require more drastic conditions such as rhabdomyolysis, hyperkalemia, loss of consciousness, or cardiopulmonary instability. These conditions require drastic monitoring and measures, including blood volume expansion, IV frusemide, mannitol, and sodium bicarbonate, and monitoring serum potassium and calcium levels [37]. 

In the current study, half of the patients (50%) were diagnosed with HS cases, and the other half were diagnosed with HE. These percentages are different from what was reported in a previous study in 2018, which showed that 29% of the cases were diagnosed with HS and 67.75% diagnosed with HE (*p* = 0.0029) [12]. The gender ratio of the study participants was almost equal, which is similar to a previous Saudi study conducted in 2016 [12]. More than half of our study sample were 60 years old or above; however, the age average in our study was higher than what was reported in another study conducted in Makkah. In our study, the average age for males and females was 60.2 and 56 years, respectively. While in a previous study, the average ages were 53.6 and 50 years for males and females, respectively [38]. The vast majority (93.1%) of HRI cases in this study occurred among pilgrims from outside Saudi Arabia, which is similar to a previous study conducted among pilgrims [12]. This could be due to the familiarity of the Saudi residents with the nature of their environment and extreme temperature rise, especially during the summer season and due to the public awareness spread throughout the Kingdom before the Hajj season [39]. 

This study has some limitations. Due to the rush hours in Hajj, there was incomplete data documentation, which made it more difficult to track patients’ data and the level of compliance in some parts of the pathway. Moreover, the case management process had gone through more than one medical team, starting from pre-hospital case management and ending with in-hospital case management. This lengthy process made it more difficult to follow-up with the adherence of HCWs’ to the pathway. Unfortunately, this study did not evaluate the specific environmental, health-related, or cultural factors that may impact the level of medical staff adherence to the Saudi guidelines. Besides, the medical records did not provide sufficient clinical data to reflect the clinical characteristics and outcomes of the patients and how these variables can influence the rate of adherence to the clinical guidelines. Future studies should assess the correlation between clinical presentation and guideline adherence, as well as how the rate of adherence affects the clinical outcomes of the patients. The lack of clinical data concerning the case-specific variations was another limitation. Although we included all patients who presented with HRIs in Mecca during the Hajj event, the small sample size in our study may limit the generalizability of our findings. The lack of data regarding the number of ineligible records is another potential limitation.

Overall, the results of the present study call for increasing HCWs’ education and awareness towards the national guidelines for HRIs management during Hajj. The Saudi authorities should develop research, education, and interdisciplinary teams of environment specialists and health experts to improve adherence to the guidelines [12]. Although barriers exist, many dissemination and implementation strategies can be planned to improve adherence to the guidelines, such as educational materials in paper form or electronic versions, and posters available where care was delivered [40]. Training and educational workshops for all medical staff should be encouraged regularly. 

## 5. Conclusions

To our knowledge, this is the first time an audit survey has been conducted on the adherence to interim HRIs guidelines. This study showed variable levels of adherence to the 2019 Saudi interim guidelines for HCWs. However, there were average to above-average adherence levels among the majority of the management themes, which reflects the proper staff knowledge and training to the guidelines. Some management themes and subthemes had poor or below-average scores, reflecting the room for further improvement in the future Hajj seasons. 

Annual audits and feedback should be planned to summarize the clinical performance of HCWs to further ensure the quality of healthcare services. Moreover, sufficient and proper documentation is essential to facilitate communication between all medical teams, evaluate the HRIs guidelines, and assess compliance levels. Training and educational workshops for all medical staff should be encouraged regularly. Additionally, more effective channels and solutions for the proper dissemination of guidelines should be taken into consideration, particularly in terms of online publication of these guidelines to the public and HCWs. 

## Figures and Tables

**Table 1:** Demographic characteristics of the enrolled population diagnosed with heat stroke (HS) and heat exhaustion (HE) in the 2019 Hajj (*n* = 98).

Variable		HE (*n*%)	HS (*n*%)	Total (*n*%)
**Age (*n* = 85)**	<30	2 (4.5%)	0	2 (2.4%)
	30–44	8 (18.2%)	5 (12.2%)	13 (15.3%)
	45–59	11 (25%)	12 (29.3)	23 (27.1%)
	≥60	23 (52.3%)	24 (58.5%)	47 (55.3%)
**Gender (*n* = 97)**	Male	25 (52.1%)	23 (46.9%)	48 (49.5%)
	Female	23 (47.9%)	26 (53.1%)	49 (50.5%)
**Residency (*n* = 87)**	Within Saudi Arabia	5 (11.1%)	1 (2.4%)	6 (6.9%)
	Outside Saudi Arabia	40 (88.9%)	41 (97.6%)	81 (93.1%)
**Nationality (*n* = 96)**	African Region	3 (6.3%)	10 (20.8%)	13 (13.5%)
	Eastern-Mediterranean Region	30 (62.5%)	15 (31.3%)	45 (46.9%)
	European Region	1 (2.1%)	3 (6.3%)	4 (4.2%)
	Region of Americas	1 (2.1%)	2 (4.2%)	3 (3.1%)
	South-East Asia Region	12 (25%)	18 (37.5%)	30 (31.3%)
	Western-Pacific Region	1 (2.1%)	0	1 (1.0%)

HS: heat stroke; HE: heat exhaustion.

**Table 2:** Heat exhaustion management guidelines compliance scores for the heat exhaustion cases (*n* = 98).

Heat Exhaustion Management Theme/Subtheme	Total Number of Eligible Cases	Number of Cases Consistent with Guidelines (*n*%)	Guideline Compliance Score
**Basic Case Management**			5.5
Patient moved to a cooler place	49	46 (93.9%)	9.3
Patient placed in supine position and elevate legs and hips	49	19 (38.8%)	3.8
Patient clothes lightened up	49	31 (63.3%)	6.3
Patient started oral hydration	49	14 (28.6%)	2.8
**Advanced Case Management**			4.7
Intravenous fluid given when patient nauseated	9	5 (55.6%)	5.5
Cooling and transferring the patient to health facility	49	21 (42.9%)	4.3
If no improvement of signs and symptoms, other diagnosis considered	49	22 (44.9%)	4.4

**Table 3:** Heatstroke pre-hospital management guidelines compliance scores (*n* = 98).

Heatstroke Management Theme/Subtheme	Total Number of Eligible Cases	Number of Cases Consistent with Guidelines (*n*%)	Guideline Compliance Score
**Pre-hospital considerations**			7.2
BSI * considered	49	40 (81.6%)	8.1
Any medication given pre-hospital	49	32 (65.3%)	6.5
Full pre-hospital report taken	49	34 (69.4%)	7.0
**Recognition of heatstroke**	49	40 (81.6%)	8.1
**Case management**			
**Case assessment**			7.7
Responsiveness assessed	49	40 (81.6%)	8.1
All vital signs documented	49	37 (75.5%)	7.4
**Stabilize ABC**			8.7
Airway stabilized	49	40 (81.6%)	8.1
Breathing stabilized	49	40 (81.6%)	8.1
Circulation stabilized	14	14 (100%)	10
**Cooling**			5
Started immediately on the scene	49	17 (34.7%)	3.6
Ice packs/chemical ice packs, fanning, wet sheets to the skin were applied	49	32 (65.3%)	6.5
Cooling continued on the way to the heatstroke unit	49	15 (30.6%)	3.0
Patient transferred immediately to the heatstroke unit	49	35 (71.4%)	7.1

* Body substance isolation is a system of infection precautions intended to reduce nosocomial transmission of infectious agents among patients (a practice of isolating all body substances (blood, urine, feces, tears, etc.)). ABC: airway, breathing, and circulation.

**Table 4:** Heatstroke in-hospital management guidelines compliance scores (*n* = 98).

Heatstroke Management Theme/Subtheme	Total Number of Eligible Cases	Number of Cases Consistent with Guidelines (*n*%)	Guideline Compliance Score
**In-Hospital Considerations**			7.6
● Adhered to appropriate PPEs	49	49 (100%)	10
● Did not give antipyretics	49	37 (75.5%)	7.5
● Full pre-hospital report taken	49	27 (55.1%)	5.5
**Diagnosis confirmation with rectal thermometer**	49	37 (75.5%)	7.5
**Case Management**			
**A.** **Cooling**			7.6
● Cooling continued	49	48 (98%)	9.7
● Skin and rectal temperature continuously monitored	49	48 (98%)	9.7
● Skin temperature maintained > 30 °C	49	17 (34.7%)	3.4
● Cooling stopped when rectal temperature is 39 °C	49	38 (77.6%)	7.7
**B.** **Stabilize ABC**			9.5
● Airway stabilized	49	48 (98%)	9.7
● Breathing stabilized: administer oxygen to keep oxygen saturation (SaO2) > 94	49	46 (93.9%)	9.3
**Special considerations**			4.3
● Rhabdomyolysis diagnosed	49	9 (18.4%)	1.8
● Volume expanded by giving more fluids	49	20 (40.8%)	4.0
● Intravenous furosemide, mannitol, and sodium bicarbonate were given	49	3 (6.1%)	0.6
● Potassium and calcium were monitored	49	23 (46.9%)	4.6
● If hyperkalaemia, the patient was treated	14	4 (28.6%)	3.0
● In case of seizure/shivering, benzodiazepines given	21	17 (81%)	8.0
● In case of multiple organ system dysfunction, supportive therapy given	7	6 (85.7%)	8.5

ABC: airway, breathing, and circulation (ABC); PPEs: Personal protective equipment.

## Data Availability

Data sharing not applicable.

## Supplementary Materials

The following are available online at https://www.mdpi.com/1660-4601/18/3/1156/s1, File S1: study’s data collection checklist.
