# Supplementary material for: Adherence of Healthcare Workers to Saudi Management Guidelines of Heat-Related Illnesses during Hajj Pilgrimage
Source: Int J Environ Res Public Health. 2021 Jan 28;18(3):1156. doi: 10.3390/ijerph18031156 (PMC7908601; doi:10.3390/ijerph18031156)
Supplement: Supplementary file 1 [file ijerph-18-01156-s001.pdf]

GLOBAL CENTER FOR MASS GATHERINGS MEDICINE

وزارة الصحة
Ministry of Health

Heat Related-Illnesses Clinical Audit in Hajj 1440H - Cross-sectional study
Data collection tool

**Label #:**	**Date of collection:** DD / MM / **2019**<br>**Name of data collector:**<br>**Name of Facility:**<br>**Time:**

**Section (1): Demographics الديموغرافيات**	
**1.1 Gender الجنس :** ☐ Male ذكر ☐ Female أنثى	**1.4 Nationality:الجنسية** ________________<br>**1.5 Country of origin:بلد المنشأ**
**1.2 Ageالعمر :** __________ years<br>**1.3 Residence of pilgrim بلد إقامة الحاج:**<br>☐ **Within KSA داخل المملكة**<br>☐ **Outside KSA خارج المملكة**	**1.6 Hospital MRN:الرقم الطبي**
**Section (2): Admission Details معلومات الدخول**	
**2.1 Patient presented at قدم إلى:**<br>☐ **Emergency الطوارئ** ☐ **Out-patient clinic العيادات الخارجية**	**2.2 Date of arrival to hospital:تاريخ القدوم للمستشفى**<br>DD / MM / **2019**<br>**Time الوقت:** ___:___ ☐ AM صباحاً ☐ PM مساءاً
**2.3 Admitted تم التنويم:** ☐ **Yes** ☐ **No**	**2.4 Admission التنويم** ☐ Ward جناح#______ ☐ ICU العناية المركزة#_____
**2.5 Date of admission تاريخ الدخول:** DD / MM / **2019**	**2.6 Date of discharge تاريخ الخروج:** DD / MM / **2019**

**Section (3) Management of suspected Heat Exhaustion ادارة حالات الاجهاد الحراري المشتبهة**			
**3.1** Patient moved to a cooler place تم نقل المريض لمكان أبرد	☐ **Yes** ☐ **No**	**3.3** Patient clothes lightened up تم تخفيف ملابس المريض	☐ **Yes** ☐ **No**
**3.2** Patient placed in supine position and elevate legs and hips تم وضع المريض على ظهره و رفع ساقية و وركيه	☐ Yes ☐ No	**3.4** Patient started oral hydration بدأ ترطيب المريض عن طريق الفم	☐ Yes ☐ No
**3.5** Patient had **NAUSEA** المريض يشتكي من الغثيان	☐ **Yes** ☐ **No**	**3.5a If YES,** Intravenous fluid given اذا نعم، تم إعطاء المريض محلول عن طريق الوريد	☐ **Yes** ☐ **No**
**3.7** Patient developed:بدء ظهور<br>**3.7a. neurologic symptoms** أعراض عصبية	☐ **Yes** ☐ **No**	**3.8 If YES اذا نعم:**<br>**3.8a.** Cooling started تم بدأ التبريد	☐ **Yes** ☐ **No**
**3.7b. increased body temperature** ارتفاع درجة حرارة الجسم	☐ **Yes** ☐ **No**	**3.8b.** Transferred quickly to a health facility (hospital) with a heatstroke unit تم نقل المريض فورا الى منشأة صحية (مستشفى) لديها وحدة علاج ضربات الحرارة	☐ **Yes** ☐ **No**
**3.7c. sustained hypotension** انخفاض مستمر في ضغط الدم	☐ **Yes** ☐ **No**		
**3.9** Signs and symptoms of heat exhaustion didn't improve لم تتحسن أعراض الاجهاد الحراري	☐ **Yes** ☐ **No**	**3.10 If No,** other diagnoses considered (refer to registry criteria below) إذا لا، هل تم اعتبار تشخيص مختلف	☐ Yes ☐ No

Section (4) Management of SUSPECTED HEATSTROKE (Prehospital settings)
إدارة حالات ضربات الحرارة المشتبهة –(قبل دخول المستشفى)

4.1 **BSI*** considered تم تطبيق معايير الصحة الوقائية للتعامل مع سوائل الجسم ☐ **Yes** ☐ **No**

*__Body substance__ isolation is a system of infection precautions intended to reduce nosocomial transmission of infectious agents among patients (a practice of isolating all **body substances** (blood, urine, feces, tears, etc)

©2019.GCMGM
HRICA19

GLOBAL CENTER FOR MASS GATHERINGS MEDICINE

وزارة الصحة
Ministry of Health

Heat Related-Illnesses Clinical Audit in Hajj 1440H - Cross-sectional study
Data collection tool

**Label #:**	**Date of collection:** DD / MM / 2019<br>**Name of data collector:**<br>**Name of Facility:**<br>**Time:**

4.2 Any medications given prehospital تم إعطاء أي أدوية قبل وصول المستشفى	☐ **Yes** ☐ **No**	4.3 Full prehospital report taken (e.g. if IVF was given, documented temperature…) تم أخذ تقرير كامل عن الحالة (إعطاء محاليل، المؤشرات الحيوية)	☐ Yes ☐ No
**4.4 Recognition of heat stroke signs** تشخيص ضربة الحرارة وفقا للعلامات			
4.4a Exposure to extreme environmental heat التعرض لحرارة جو شديدة	☐ **Yes** ☐ **No**	4.4b Mental Status alteration تغيرات في الحالة العقلية	☐ **Yes** ☐ **No**
4.4c Core Body **temperature > 40°C** درجة حرارة الجسم المركزية > ٤٠	☐ Yes ☐ No	4.4d Other differential diagnosis considered تم اعتبار تشخيص مختلف	☐ Yes ☐ No
4.8 **REMOVED** from heat and lightened up clothes تم نقلة من مكان الحرارة وتخفيف الملابس			☐ **Yes** ☐ **No**
4.9 **Responsiveness** (AVPU*) assessed تقييم حالة الوعي والاستجابة<br>*AVPU scale (an acronym from "alert, verbal, pain, unresponsive")	☐ **Yes** ☐ **No**	4.10 All Vital signs documented (TEMP, HR, SO2, GLUCOSE, BP) تم تسجيل وكتابة المؤشرات الحيوية	☐ **Yes** ☐ **No**
**4.11 STABILIZE (ABCs) استقرار مجرى الهواء والتنفس والدورة الدموية:**			
**4.11a. AIRWAY stabilized** تم استقرار و فتح مجرى الهواء	☐ **Yes** ☐ **No**	**4.11b. BREATHING stabilized** administer oxygen to **keep SaO2 >94%** تم استقرار التنفس (نسبة الاوكسجين >٩٤%)	☐ **Yes** ☐ **No**
**4.11c. CIRCULATION stabilized** تم استقرار الدورة الدموية<br>- Normal saline 500 ml given تم إعطاء محلول ملحي ٥٠٠ مل<br>- Patient is still hypotensive (expand blood volume and manage hypotension) and more fluids given (500 ml Normal Saline) مازال المريض يعاني من انخفاض ضغط الدم (تم إعطائه المزيد من المحاليل+٥٠٠مل)			☐ **Yes** ☐ **No**<br>☐ **Yes** ☐ **No**
**4.12 COOLING التبريد:**			
4.12a. Started immediately on scene تم التبريد فورا في الموقع	☐ **Yes** ☐ **No**	4.12b. Ice packs/chemical ice packs, fanning, wet sheets to the skin were applied تم التبريد باستخدام عبوات ثلج/عبوات ثلج كيميائية، تهوية، شراشف مبللة على الجلد	☐ **Yes** ☐ **No**
4.12c. Cooling continued on the way to heatstroke unit تم استمرار عملية تبريد في الطريق الى وحدة ضربات الحرارة	☐ **Yes** ☐ **No**	4.12d. Patient **transferred** immediately to heatstroke unit تم نقل المريض فوراً لوحدة ضربات الحرارة	☐ Yes ☐ No
**Section (5) Management of Suspected Heatstroke**<br>**(In-hospital settings: In the heatstroke unit)** (داخل المستشفى – وحدة ضربات الحرارة) **إدارة ضربات الحرارة المشتبهة**			
5.1 Adhered to appropriate PPEs تم تطبيق إجراءات الصحة الوقائية اللازمة و وضع معدات الحماية الشخصية اللازمة	☐ **Yes** ☐ **No**	5.2 Antipyretics given تم إعطاء خافض للحرارة	☐ **Yes** ☐ **No**
5.3 Full prehospital report taken (e.g. if IVF was given, documented temperature…) تم اخذ التقرير الكامل لقبل وصول المريض للمستشفى			☐ **Yes** ☐ **No**
5.4 **Diagnosis confirmed** with **rectal thermometer** calibrated to measure high temperatures to **>42°C** تم تأكيد التشخيص عن طريق قياس الحرارة بميزان حرارة شرجي	☐ **Yes** ☐ **No**	5.5 Mental Status alteration تغيرات في الحالة العقلية	☐ **Yes** ☐ **No**

©2019.GCMGM
HRICA19

GLOBAL CENTER FOR MASS GATHERINGS MEDICINE

وزارة الصحة
Ministry of Health

Heat Related-Illnesses Clinical Audit in Hajj 1440H - Cross-sectional study
Data collection tool

Label #:	Date of collection: DD / MM / 2019<br>Name of data collector:<br>Name of Facility:<br>Time:

**5.6 COOLING:التبريد**			
5.6a. Cooling **continued** استمرار التبريد	☐ Yes ☐ No	5.6b. Skin and rectal temperature was continuously **monitored** or every 10 minutes استمرار قياس الحرارة الجلد وميزان الحرارة الشرجي او كل ١٠ دقائق	☐ Yes ☐ No
5.6c. Skin temperature **maintained >30℃** الحفاظ درجة حرارة الجلد > ٣٠	☐ Yes ☐ No	5.6c. Cooling **stopped** when rectal temperature is **39℃** تم التوقف عن التبريد عند وصول الحرارة الشرجية ٣٩	☐ Yes ☐ No
5.7 **Responsiveness** (AVPU*) assessed and documented تقييم وتسجيل حالة الوعي والاستجابة<br>*AVPU scale (an acronym from "alert, verbal, pain, unresponsive")	☐ Yes ☐ No	5.8 All Vital signs documented (TEMPERATURE, HR, SpO2, GLUCOSE, BP) تسجيل وكتابة جميع المؤشرات الحيوية	☐ Yes ☐ No
**5.9 STABILIZE (ABCs):استقرار مجرى الهواء والتنفس والدورة الدموية**			
**5.9a. AIRWAY stabilize**تم استقرار و فتح مجرى الهواء : Patient intubated تم وضع أنبوب تنفسي	☐ Yes ☐ No	**5.9b. BREATHING stabilized** administer oxygen to **keep SaO2 >94** تم استقرار التنفس (نسبة الاوكسجين >٩٤%)	☐ Yes ☐ No
**5.10 Other considerations اعتبارات أخرى:**			
**5.10a. Rhabdomyolysis diagnosed: تم تشخيص انحلال الربيدات (العضلات)**			☐ Yes ☐ No
5.10a1. Volume expanded by giving more fluids تم إعطاء محاليل بالوريد	☐ Yes ☐ No	5.10a2. Intravenous **furosemide, mannitol** and **sodium bicarbonate** was given تم إعطاء ادوية مثل فروسامايد و مانيتول و بيكربونات الصوديوم عن طريق الوريد	☐ Yes ☐ No
5.10a3. Potassium and calcium was **monitored** مراقبة مستويات البوتاسيوم والكالسيوم بالدم	☐ Yes ☐ No	5.10a4. If hyperkalemia; **treated** في حال وجود انخفاض في مستوى البوتاسيوم بالدم، هل تم علاجه؟	☐ Yes ☐ No
**5.10b. Seizure/shivering التشنجات/الرعاش:**			☐ Yes ☐ No
5.10b1. Benzodiazepines given (ادوية للتشنج) تم إعطاء بينزوديازبين			☐ Yes ☐ No
**5.11 Post cooling** بعد التبريد<br>- Multiple organ system dysfunction ضرر في وظائف أعضاء متعددة بالجسم<br>- Supportive therapy given تم إعطاء علاج دعم			☐ Yes ☐ No<br>☐ Yes ☐ No

©2019.GCMGM
HRICA19
